# The effects of aging on fear learning in male mice

**DOI:** 10.1093/geroni/igaf122.4025

**Published:** 2025-12-31

**Authors:** Chad Smies, Jiyeon Baek, Janine Kwapis

**Affiliations:** The Pennsylvania State University, University Park, Pennsylvania, United States; The Pennsylvania State University, University Park, Pennsylvania, United States; The Pennsylvania State University, University Park, Pennsylvania, United States

## Abstract

Cognitive decline is prevalent in the aging population, in both human and rodent models. Mice show age-related impairments in fear learning and dysregulated gene expression following a learning event. While many studies investigate physical decline in aging, little is known about how maladaptive learning observed in post-traumatic stress disorder (PTSD) manifests in the aging population. The current study aims to characterize and identify intervention points to support appropriate fear learning with age. Fear conditioning is commonly used to model PTSD in rodents, and memory is measured as freezing behavior. Contextual fear conditioning (CFC) pairs a shock with a specific context. In contrast, delay (DFC) and trace fear (TFC) conditioning pair a shock that either co-terminates with a cue or is presented after a trace interval, respectively. Extinction is commonly used as a treatment model for PTSD, where the cue or context is repeatedly presented in the absence of a shock. Recent work has revealed age-associated deficits in CFC extinction and TFC using an auditory cue. The current study characterizes fear responses in TFC and DFC and extinction in aged mice using a novel light cue that can be presented outside the conditioning chamber. We found subtle, but reliable, deficits in freezing behavior in aged mice. Importantly, the successful pairing of the shock with the light cue allows future studies to examine brain activity while presenting a shock-associated light cue with functional magnetic resonance imaging (fMRI) to determine activity changes that occur in PTSD with aging, providing insight for future treatments.

